# Managing Refractory Pericarditis in the Era of Biologic Therapy: A Cardio-Oncology–Based Risk Framework

**DOI:** 10.1016/j.jacadv.2026.102781

**Published:** 2026-05-14

**Authors:** Jaideep Singh Bhalla, Ushasi Saraswati, Allan L. Klein

**Affiliations:** a Department of Cardiovascular Medicine, Montefiore Medical Center, Bronx, New York, USA; b Center for Diagnosis and Treatment of Pericardial Diseases, Section of Cardiovascular Imaging, Department of Cardiovascular Medicine, Heart, Vascular and Thoracic Institute, Cleveland Clinic, Cleveland, Ohio, USA

**Keywords:** cardio-oncology, genetics, interleukin-1, malignancy risk, recurrent pericarditis

**Figure:**
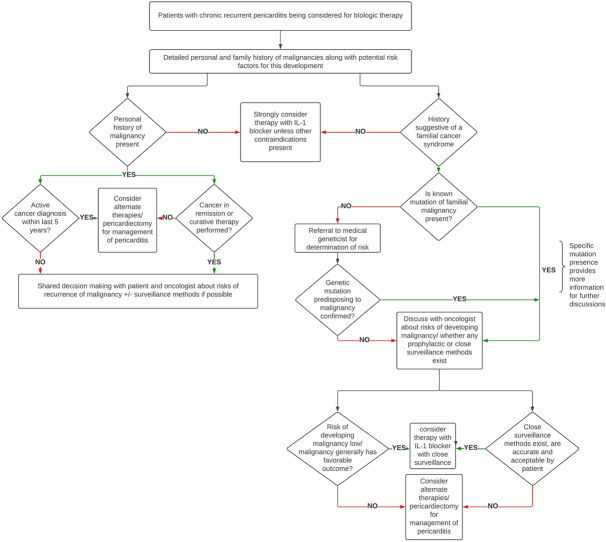

Chronic and recurrent pericarditis (RP) is a disease marked by high morbidity, frequent relapses, impaired quality of life, and cumulative treatment toxicity. Although conventional therapies—primarily steroids, nonsteroidal anti-inflammatory drugs, and colchicine—have proven effective, they are not without drawbacks; particularly corticosteroids, which carry a substantial risk of adverse effects.^1^ The emergence of interleukin (IL)-1 α/β inhibition has transformed the therapeutic landscape for refractory RP, offering rapid symptom control and sustained remission with encouraging short-term safety data.^2^ However, as IL-1 inhibitors are increasingly used for prolonged durations in patients who remain therapy-dependent, important questions regarding long-term oncologic safety have emerged. Because IL-1 signaling intersects with pathways central to tumor biology and immune surveillance, concerns persist regarding the potential risk of malignancy, particularly in individuals with pre-existing cancer susceptibility or prior neoplastic history.^3^ Clinicians are therefore confronted with a complex decision: how to balance the need for durable inflammatory control and steroid sparing against the theoretical—and incompletely defined—risk of malignancy in a growing population of at-risk patients.

As illustrated in a prior case-based discussion of RP in a patient with significant oncologic risk factors, individualized decision-making in this setting can be particularly challenging and highlights the limitations of the existing evidence.^4^ Despite reassuring short-term safety data, uncertainty persists regarding the long-term oncologic implications of sustained IL-1 inhibition. Although isolated malignancies have been reported with prolonged biologic use—particularly with agents such as anakinra—randomized trials of rilonacept have not demonstrated a definitive causal association.^5^, ^6^, ^7^ Importantly, these studies were not powered to evaluate malignancy risk, and follow-up durations remain limited. Moreover, pivotal trials excluded patients with recent cancer diagnoses, further constraining generalizability to individuals at elevated oncologic risk. As a result, clinicians are frequently left navigating incomplete evidence when managing patients with prior malignancy, strong family histories, or suspected genetic predisposition. The challenge is not simply estimating theoretical cancer risk, but determining how to balance that uncertainty against the substantial morbidity of uncontrolled pericarditis and the harms of chronic corticosteroid exposure, or operative risks and lifelong consequences of an early radical pericardiectomy. In this setting, a structured, multidisciplinary approach becomes essential—one that protects against unnecessary oncologic risk while avoiding reflexive denial of highly effective IL-1–directed therapy.

In light of these uncertainties, we propose a structured pathway to guide shared decision-making for patients with refractory RP being considered for IL-1–directed therapy (Figure 1). The framework begins with a deliberate assessment of oncologic risk, including a detailed personal history of malignancy, a comprehensive family history, and evaluation for features suggestive of hereditary cancer syndromes. A thorough evaluation before the initiation of biologic therapy is essential to identify these individuals at increased risk, and referral to medical genetics and coordination with oncology are essential to clarify individualized risk. This may be achieved via the use of validated risk calculators^8^^,^^9^ in specific malignancies, as well as by incorporating strategies for prevention such as close surveillance or definitive surgery, when deemed appropriate. This approach could similarly be adapted for patients with a history of prior malignancy who are in remission, ensuring that both the underlying inflammatory condition and potential cancer risks are managed in tandem. The management of patients with active or recent malignancy—particularly within 5 years—remains especially complex given their exclusion from pivotal RP trials^6^^,^^7^; in these cases, transparent discussion of evidentiary limitations is critical, with decisions individualized according to cancer prognosis, inflammatory burden, and patient values. Importantly, this pathway is not intended to create reflexive barriers to IL-1 therapy, but rather to contextualize its use within a structured risk–benefit assessment. When oncologic risk is deemed low or mitigable through surveillance, IL-1 inhibition may proceed with appropriate monitoring. Conversely, when cancer risk is substantial or surveillance strategies are inadequate, alternative approaches—including surgical pericardiectomy—may warrant consideration. Finally, clinicians should remain vigilant for the possibility that pericarditis itself may represent a paraneoplastic or early malignant manifestation. This possibility warrants particular consideration in patients with atypical clinical features, refractory disease patterns, or systemic inflammatory phenotypes—especially those with autoimmune or immune-mediated etiologies, where heightened immune activation and exposure to adjunct immunosuppressive therapies may further modify oncologic risk.^10^ Clinicians must remain vigilant for clinical signs and laboratory findings that could suggest an occult malignancy and utilize targeted diagnostic modalities, such as advanced imaging or biomarker testing, to aid in their timely diagnosis.

**Figure 1:**
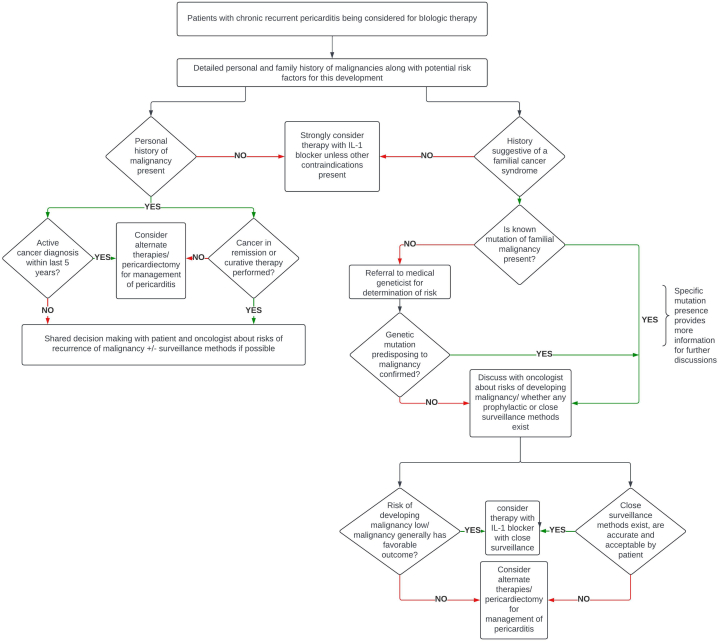
**Algorithm for Evaluation of Interleukin-1 Therapy in Recurrent Pericarditis With Elevated Oncologic Risk** Proposed clinical algorithm to guide structured oncologic risk stratification in patients with chronic recurrent pericarditis (RP) being considered for interleukin (IL)-1 inhibition. The pathway begins with comprehensive assessment of personal malignancy history, timing of prior cancer, and family history suggestive of hereditary cancer syndromes. Key decision nodes delineate scenarios that may represent relative contraindications to biologic therapy and identify areas of evidentiary uncertainty requiring multidisciplinary discussion with oncology and medical genetics. When malignancy risk is low or can be mitigated through surveillance or preventive strategies, IL-1 blockade with close monitoring is appropriate; when risk is substantial or not modifiable, alternative therapy or pericardiectomy should be considered. Diamonds indicate decision nodes; rectangles denote management steps; green arrows represent affirmative pathways; red arrows represent negative pathways.

In conclusion, IL-1–directed biologic therapy has transformed the management of refractory RP, but its expanding use requires a parallel evolution in how we assess and contextualize oncologic risk. Rather than defaulting to avoidance in patients with heightened cancer susceptibility, clinicians should adopt a structured, multidisciplinary framework that integrates individualized risk assessment, transparent discussion of evidentiary limitations, and coordinated surveillance strategies. Such an approach enables precision in decision-making—preserving access to highly effective, steroid-sparing therapy while responsibly addressing potential malignancy concerns. Ultimately, we hope that this proposed personalized care model will help optimize outcomes while minimizing potential harms in this challenging patient population. Future prospective registry data and collaborative cardio-oncology research efforts are urgently needed to better define long-term cancer risk across pericarditis subtypes, develop validated risk stratification tools, and clarify optimal treatment duration when malignancy emerges during IL-1 inhibition. As biologic therapies become increasingly embedded in inflammatory cardiovascular care, the intersection between immune modulation and cancer risk will demand continued vigilance, rigorous study, and thoughtful clinical leadership.

## Funding support and author disclosures

Dr Klein has received research grants and scientific advisory board from Kiniksa Therapeutics and Cardiol Therapeutics during the conduct of the study. The authors have reported that they have no relationships relevant to the contents of this paper to disclose.
